# A Rare Case of Superior Mesenteric Artery Thrombosis With Gangrenous Bowel in a Patient With Mitral Valve Replacement in a Hypocoagulable State

**DOI:** 10.7759/cureus.73731

**Published:** 2024-11-15

**Authors:** Prithvinathan Vasudevan, Raghupathy Thirunavikkarasu, Magesh Chandran, Madan Sundar

**Affiliations:** 1 General Surgery, Sree Balaji Medical College and Hospital, Chennai, IND

**Keywords:** acute mesenteric ischemia (ami), bowel gangrene, ischemic enteritis, mechanical mitral valve replacement, mechanical prosthetic valve thrombosis, resection and anastomosis, superior mesenteric artery thrombosis

## Abstract

Acute thromboembolism of the superior mesenteric artery (SMA) causing mesenteric ischemia has a grave prognosis with high mortality rates. Its rarity and non-specific symptoms often lead to delayed diagnosis and increased morbidity. Early signs can include pain out of proportion to physical findings, with abdominal distension, tenderness, and guarding appearing only in later stages when bowel necrosis has occurred. Leukocytosis, metabolic acidosis, and lactic acidosis may also be present. Immediate treatment through radiological investigation and restoration of blood flow can be through interventional radiology or surgery. In cases of infarction, resection and exteriorization are needed. Patients with mechanical mitral valves, even on blood thinners, remain at risk for thromboembolism and may benefit from novel oral anticoagulants requiring less frequent monitoring. Regular monitoring of prothrombin time and international normalized ratio (INR) is essential for those on warfarin, as drugs and dietary changes (eating green leafy vegetables) can significantly affect INR levels. Early suspicion and prompt treatment are vital for improving outcomes.

## Introduction

Acute thromboembolism of the superior mesenteric artery (SMA) causes mesenteric ischemia and bowel necrosis, which carries a grave prognosis. Mesenteric ischemia results from compromised blood flow to the intestines, arising from various causes. Embolic events (40-50%) include atrial fibrillation, myocardial infarction, valvular heart disease, and cardiac aneurysm. Thrombotic events (30-40%) encompass atherosclerosis, vasculitis, fibromuscular dysplasia, trauma, and thrombosis of pre-existing stenosis. Venous thromboembolism (10-20%) involves deep vein thrombosis, portal vein thrombosis, and mesenteric vein thrombosis, often linked to hypercoagulable states. Non-occlusive causes include hypotension, cardiac failure, sepsis, vasospasm, and medication-induced (cocaine). Rare causes comprise congenital anomalies, tumors, inflammatory bowel disease, diverticulitis, and pancreatitis.

This rare condition presents as an acute abdomen, requiring immediate attention. Delayed treatment significantly increases patient morbidity and mortality. The high mortality rate, ranging from 59% to 93% [1,2], may be due to the nonspecific signs and symptoms.

The classical teaching that pain is out of proportion to physical examination findings, where the abdomen appears soft and nontender in the early stages of acute mesenteric ischemia (AMI), often misleads clinicians, contributing to increased morbidity and mortality. Abdominal distension, tenderness, and guarding typically present in later stages, after bowel necrosis has occurred. These patients often exhibit leukocytosis, metabolic acidosis, and lactic acidosis [1,3].

Early diagnosis through radiological investigations and prompt treatment, restoring blood flow by interventional radiology and by surgical intervention, can significantly improve the patient prognosis [1].

## Case presentation

A 62-year-old female presented with a four-day history of intermittent lower abdominal pain, primarily localized to the umbilical region. The pain was accompanied by vomiting, which contained food particles and was non-bilious and non-hemorrhagic. Additionally, she experienced loose stools and melena. There was no history of hematemesis, fever, dysuria, or abdominal trauma.

Past medical and surgical history

The patient had a known history of type 2 diabetes mellitus and was on regular medications. Her past surgical history included puerperal sterilization (1991) and mitral valve replacement (1994 and 2011) with metallic valve implantation (2011).

Drug history

The patient was on nicotinamide (acenocoumarol) 2 mg orally once daily and metformin 500 mg orally twice daily. On examination, the patient was conscious, oriented, and afebrile. She exhibited no signs of pallor, icterus, cyanosis, clubbing, pitting pedal edema, or bruising and was moderately built and nourished. Vital signs included a pulse rate of 110/min, blood pressure of 150/70 mmHg, and oxygen saturation of 99% on 4L oxygen. Abdominal examination revealed lower abdominal distension with upward displacement of the umbilicus, a well-healed 2 cm scar below the umbilicus, and tenderness in the left iliac fossa and hypogastric region without a palpable mass. Bowel sounds were notably absent.

**Figure 1:**
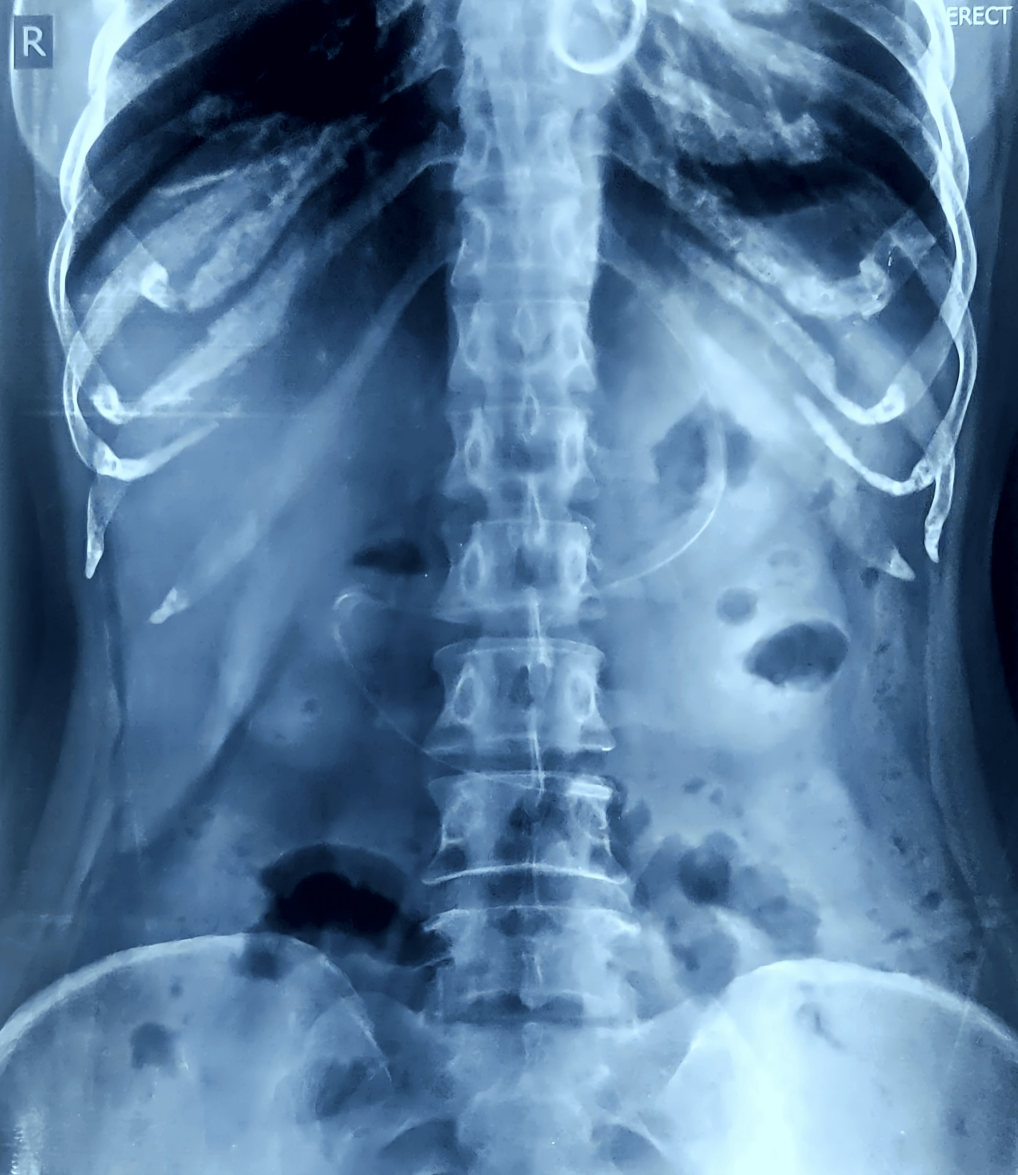
X-ray abdomen erect - normal study

**Figure 2:**
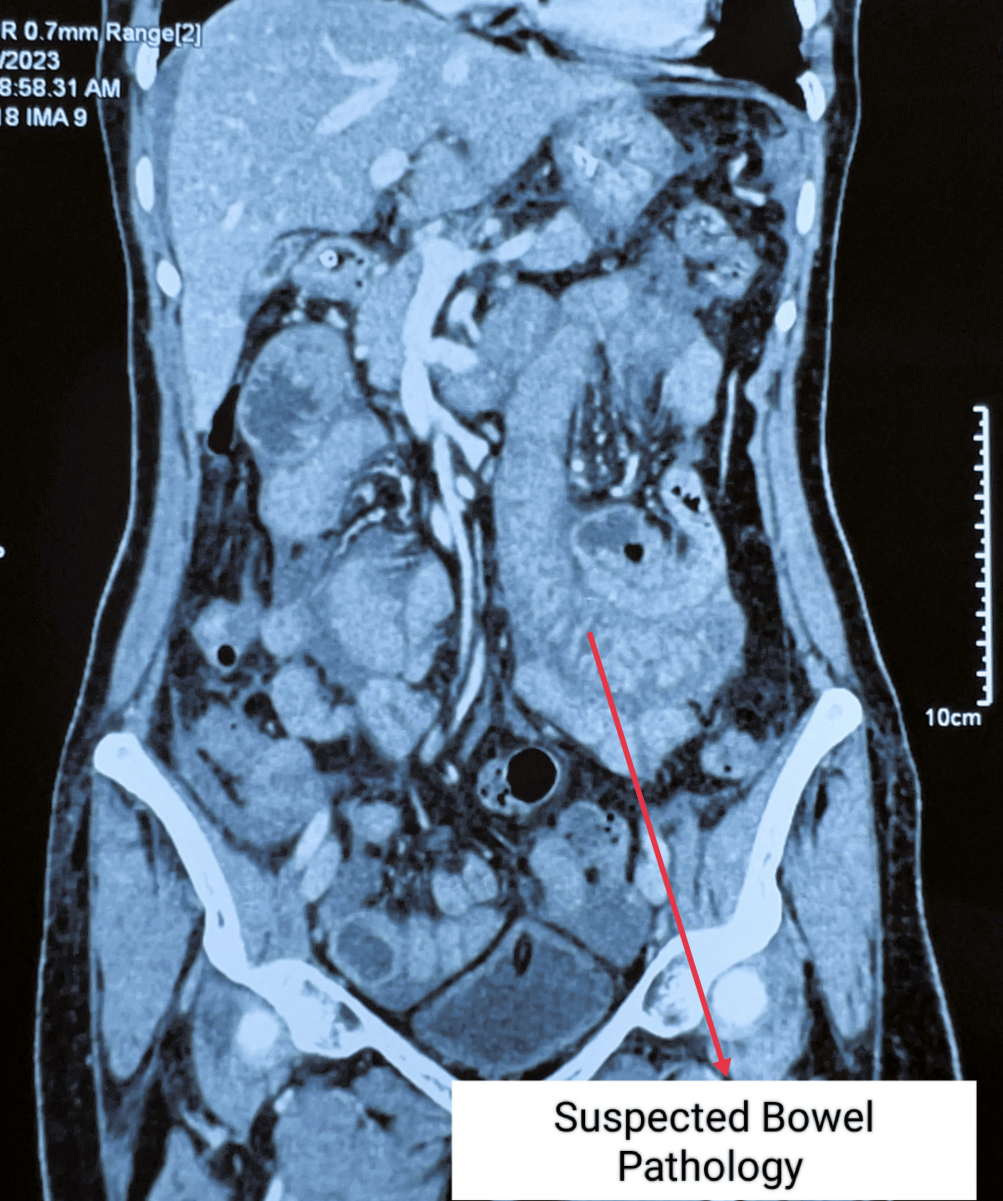
CECT of the abdomen showing suspected bowel pathology CECT: contrast-enhanced computed tomography

**Figure 3:**
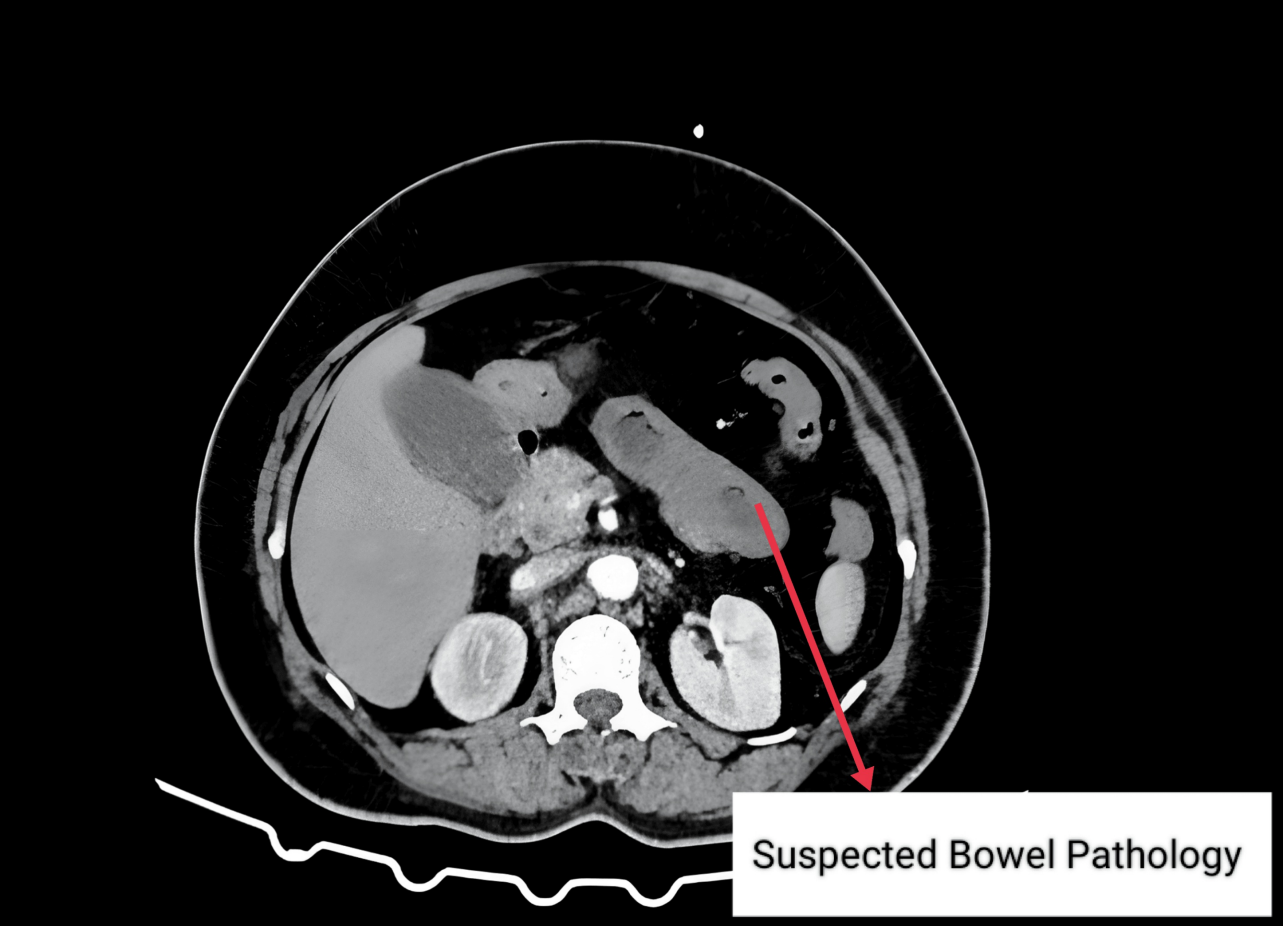
CECT abdomen showing suspected bowel pathology CECT: contrast-enhanced computed tomography

Routine blood investigations revealed hemoglobin of 10.8 g/dL, elevated total white blood cell count (19,340 cells/μL) with neutrophilia (83.1%), and electrolyte imbalances (sodium 124.8 mmol/L, potassium 3.92 mmol/L, chloride 89.7 mmol/L). Protein profile showed total protein 6.3 g/dL, albumin 3.5 g/dL, and globulin 2.8 g/dL. Arterial blood gas analysis indicated acidosis (pH 7.263, bicarbonate 14.1 mmol/L, base excess -12.9 mmol/L) with elevated lactate (5.80 mmol/L). The international normalized ratio (INR) was significantly elevated at 8.68. An erect abdominal X-ray showed no abnormalities (Figure 1). Contrast-enhanced computed tomography (CECT) of the abdomen revealed long-segment circumferential edematous thickening of jejunal loops, predominantly in the left lumbar region, with mild variable mucosal enhancement. No obvious bowel dilatation was observed, but the high-density mild-free fluid was present in the perihepatic, perisplenic, interbowel, and pelvis pouch regions, suggesting hemoperitoneum. With the possibility of spontaneous intramural small bowel hematoma with hemoperitoneum (Figures 2-3).

**Figure 4:**
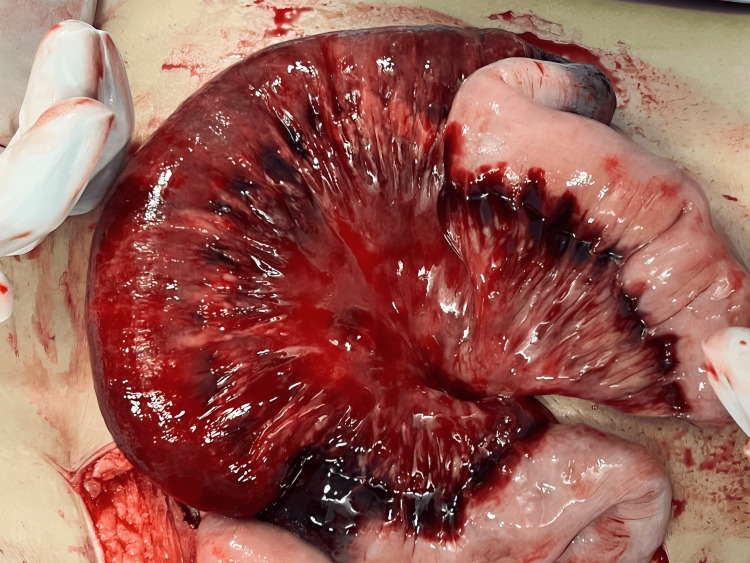
Gangrenous bowel

**Figure 5:**
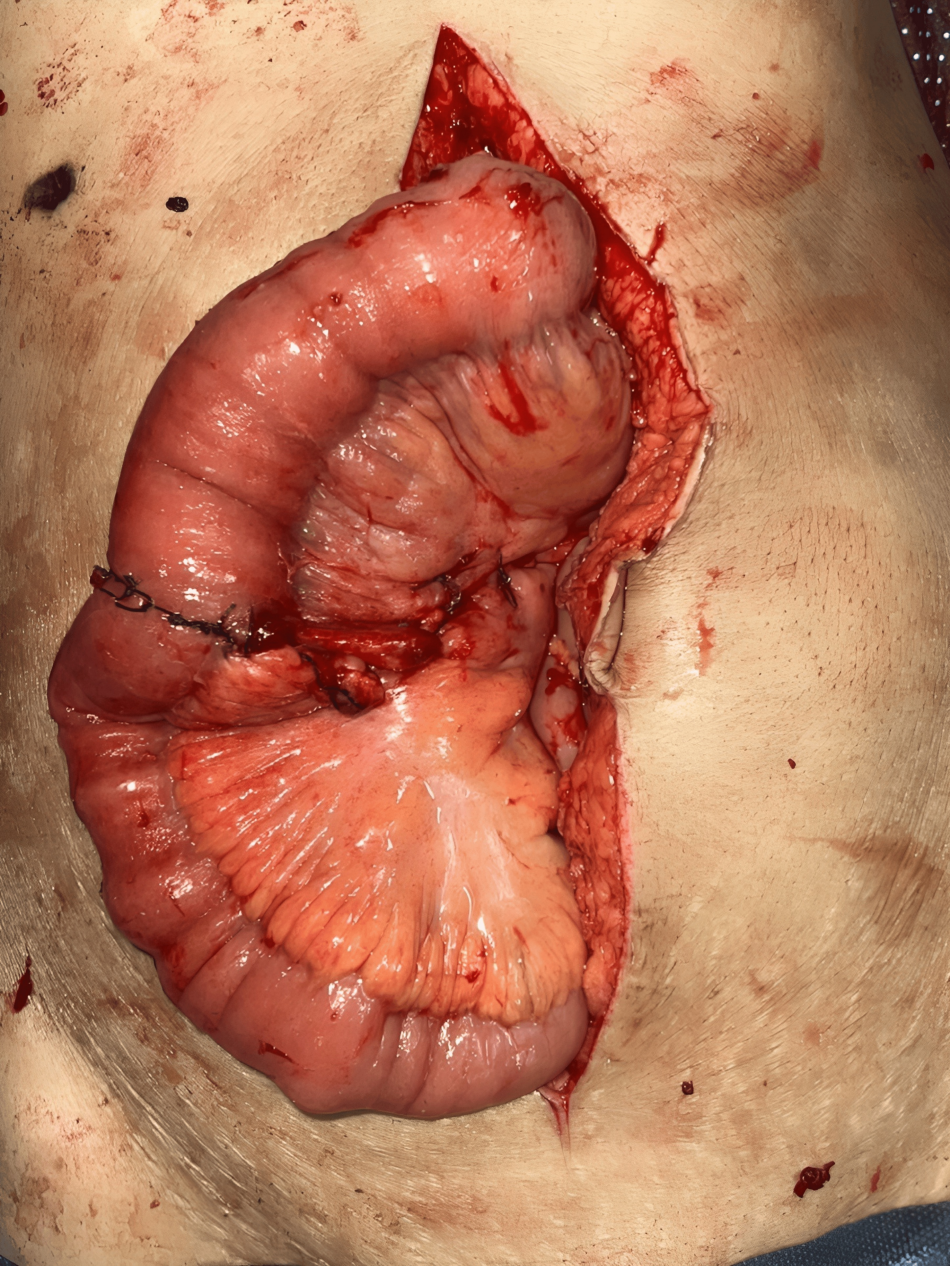
Post resection anastomosis

**Figure 6:**
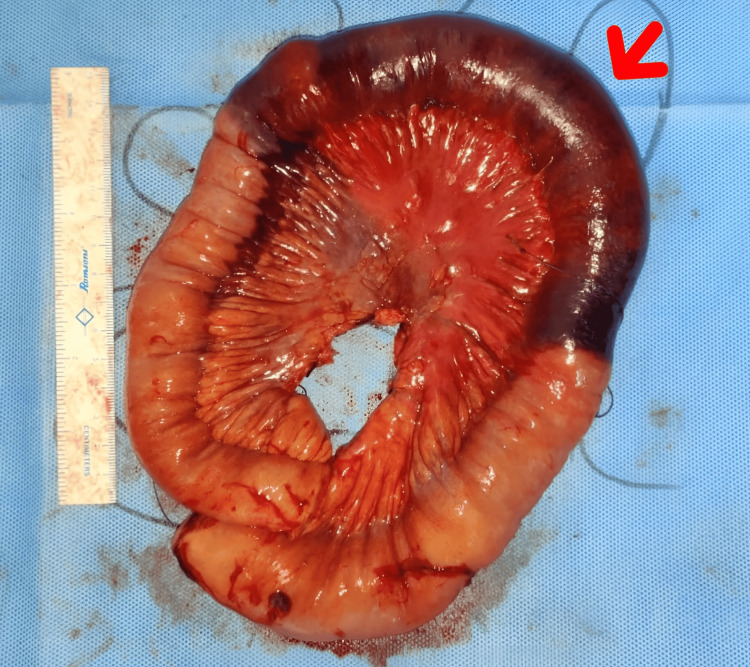
Resected bowel segment

The patient was kept nil per oral with continuous ryles tube aspiration and initiated on IV fluids, IV meropenem (1 g TDS), and IV clindamycin (600 mg BD). Metabolic acidosis correction was started. Oxygen therapy was started at 4L/min via face mask. The patient was catheterized and urine output was monitored. Additionally, 8 units of fresh frozen plasma were administered, successfully reducing the INR to 1.53. An emergency exploratory laparotomy was performed. Approximately 300 ml of hemoperitoneum was present upon opening the abdomen. Gangrenous bowel was identified 50 cm from the duodenojejunal flexure. The bowel proximal to the gangrenous part was edematous and dilated, with non-viable mesentery attachment (Figure 4). Resection and anastomosis of the gangrenous part of the bowel were performed (Figure 5), removing approximately 60 cm, including 10 cm proximally and distally from the gangrenous segment (Figure 6). End-to-end anastomosis of the jejunum was done by hand-sewn bowel anastomosis technique.

The patient was transferred to the intensive care unit (ICU) postoperatively for close monitoring. Total parenteral nutrition was initiated on postoperative day (POD) 1 and continued for five days, ensuring optimal nutrition during the initial recovery phase. The patient passed flatus on POD 3 and stools on POD 5. Oral intake was gradually introduced, starting with sips of water on POD 5, followed by clear liquids on POD 6, a semisolid diet on POD 8, and a soft, solid, bland diet on POD 10. This progression was well tolerated. Thromboprophylaxis consisted of low-molecular-weight heparin 40 mg subcutaneously once daily from POD 2 to 8 and then replaced by rivaroxaban 10 mg orally once daily. The patient was discharged on POD 16, continuing on a soft, solid bland diet for one month before progressing to a normal diet. At the six-month follow-up, the patient remained asymptomatic without complications.

Histopathology

**Figure 7:**
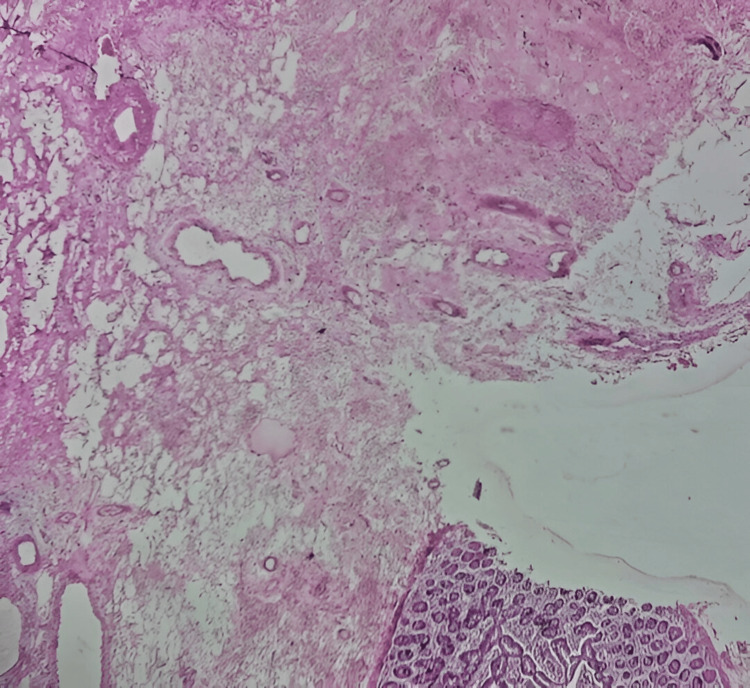
Section examined showing sloughing of mucosal areas and areas of submucosal fibrosis

**Figure 8:**
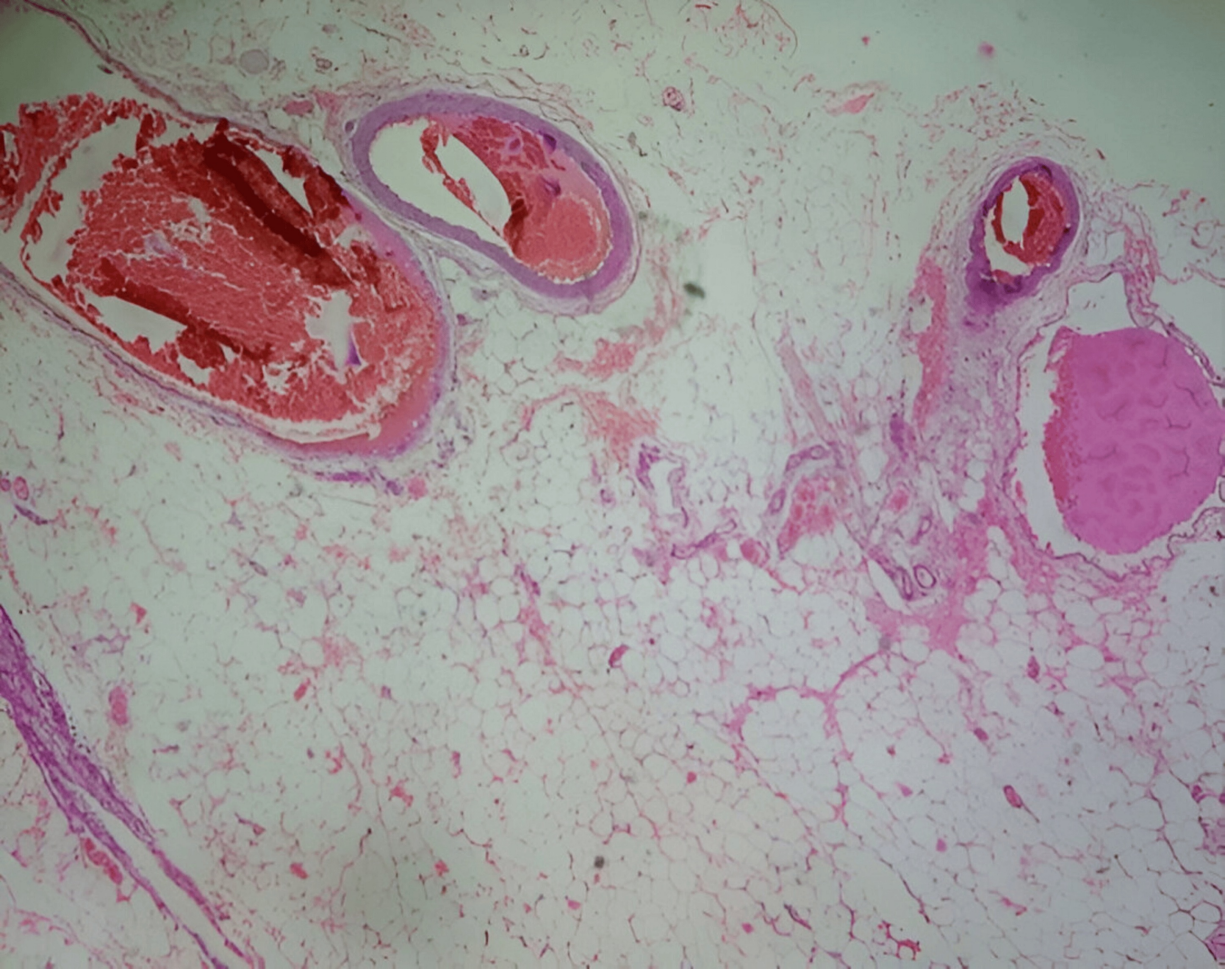
Mesenteric blood vessels showing thickening and fresh thrombi and few of them are organizing

Initial sections examined from the specimen show histological features of gangrene small intestine with areas of perforation and serosal reaction. The margins are viable. Additional sections examined show sloughing of mucosal areas and areas of submucosal fibrosis (Figure 7). Focal serosal fibrosis was also noted. The mesenteric blood vessels show thickening and fresh thrombi, and few of them are organized (Figure 8). The mesentery also shows hemorrhage and chronic inflammation. Histological features are consistent with gangrene jejunum secondary to ischemic enteritis. The margins are viable.

## Discussion

AMI is a rare but life-threatening condition that often necessitates extensive intestinal resection, leading to high mortality and potential short bowel syndrome in survivors [3,4]. It is primarily caused by compromised blood supply to the intestines, often due to thromboembolic events. Early diagnosis is essential for improving outcomes, but this is challenging due to nonspecific symptoms and laboratory findings, leading to delays in recognition [5]. Diagnostic tools such as Doppler ultrasound, angiography, computed tomography angiography, and magnetic resonance angiography are useful for identifying AMI [6,7]. Among these investigations, CT of the abdomen and pelvis with IV contrast (portal phase) remains the gold standard investigation for AMI.

In cases where there are no immediate indications for surgery, such as peritonitis or gastrointestinal hemorrhage, angiography is often the preferred approach as it can differentiate between thrombotic and nonthrombotic causes of ischemia and allow for blood flow restoration through endovascular techniques like percutaneous transluminal angioplasty or thrombolysis. If bowel necrosis has occurred, surgical resection is required.

In this case, the short segment of gangrene in the case of SMA thrombosis is likely due to the nature of the blood supply in the intestines. The SMA provides blood flow to a specific area, and when a thrombus obstructs it, only the segment directly affected by that branch may develop ischemia and gangrene. This contrasts with more diffuse ischemic conditions, where multiple vessels might be involved. So, the gangrene remains confined to the region supplied by the specific part of the artery affected.

The short segment of gangrene in cases of SMA thrombosis likely arises due to the nature of the blood supply in the intestines. The SMA provides blood flow to a specific area, and when a thrombus obstructs it, only the segment directly affected by that branch may develop ischemia and gangrene. This contrasts with more diffuse ischemic conditions, where multiple vessels might be involved. So, the gangrene remains confined to the region supplied by the specific part of the artery affected.

AMI is treated by early diagnosis, removal of necrotic bowel tissue, restoration of blood flow, and ICU care. Both thrombotic and nonthrombotic causes of bowel ischemia significantly affect the survival of the patient. The various nonthrombotic causes of bowel ischemia are low-flow states such as cardiogenic shock, pancreatitis, sepsis, hypovolemia, strangulated hernia, intussusception, trauma, and aortic dissection. Thrombotic causes include arterial embolism, arterial thrombosis, and mesenteric vein thrombosis.

Patients with AMI should be treated aggressively to increase their survival. Survival is approximately 50% when diagnosis occurs within 24 hours after onset of symptoms, but it drops sharply to 30% or less when diagnosis is delayed [8]. These patients should be treated with fluid resuscitation, hemodynamic monitoring, prophylactic antibiotics, and systemic anticoagulation drugs. In the case of mesenteric vein thrombosis, the patient should be started on injection heparin to prevent the progression of the thrombus. And should be started on long-term oral anticoagulants like warfarin to prevent recurrence. This patient should be closely monitored for fluid imbalance, as bowel congestion from venous thrombosis can lead to fluid sequestration, hemoconcentration, hypovolemia, and shock [5].

In cases of bowel infarction, the patient must undergo surgery. In arterial causes of AMI, urgent surgery is required to restore blood flow to the ischemic bowel and remove the infarcted tissue, which may involve extracting the thrombus or embolus or bypassing the blockage, and the patient has to undergo the procedure as soon as possible. The patient who underwent a procedure less than 24 hours after the onset of symptoms carries a 50% survival rate, but it drops sharply to 30% or less when treatment is delayed [8]. A conservative approach is preferred to preserve as much viable intestine as possible. If bowel viability is uncertain, a second-look laparotomy is commonly performed. Bowel viability is evaluated through visual inspection, Doppler signals, and fluorescein uptake under ultraviolet light to ensure the safe removal of nonviable tissue during the follow-up surgery.

Prosthetic valve thrombosis (PVT) is a serious complication of heart valve replacement, associated with significant morbidity and mortality. The risk is greater with mechanical valves compared to biological ones [9]. All foreign objects, including prosthetic valves, introduced into the cardiovascular system are thrombogenic, often requiring anticoagulation therapy to reduce the risk of thrombosis and prevent complications like stroke. PVT involves the formation of a thrombus on the prosthetic valve structures, leading to valve dysfunction and, in some cases, thromboembolism [10].

## Conclusions

Patients who have undergone mechanical mitral valve replacement remain at risk for thromboembolism, even while on blood thinners. These patients should be considered for direct oral anticoagulants that do not require frequent monitoring. For those on warfarin, regular monitoring of prothrombin time (PT) is essential, as PT and INR levels can fluctuate significantly with the intake of green leafy vegetables, potentially leading to complications. AMI should be suspected early in such cases, as prompt treatment is crucial for improving prognosis and reducing morbidity and mortality.
